# Tropical Infections Induced Fulminant Hepatitis in Peripartum Managed Successfully: Tales of Fate

**DOI:** 10.7759/cureus.22223

**Published:** 2022-02-15

**Authors:** Surekha Tayade, Sparsh Madaan, Sunil Kumar, Dhruv Talwar, Arzoo Chadha

**Affiliations:** 1 Department of Obstetrics and Gynaecology, Jawaharlal Nehru Medical College, Datta Meghe Institute of Medical Sciences (Deemed to be University), Wardha, IND; 2 Department of Medicine, Jawaharlal Nehru Medical College, Datta Meghe Institute of Medical Sciences (Deemed to be University), Wardha, IND

**Keywords:** tropical diseases, pregnancy, leptospirosis, dengue, malaria

## Abstract

Tropical diseases such as malaria, dengue, intestinal helminths, schistosomiasis, leishmaniasis, and filariasis have an essential influence on the reproductive health of patients.

Various cases of pregnancy loss in unexplained circumstances are a result of underdiagnosed tropical diseases. Term pregnancy complicated by tropical diseases is a challenge for the treating clinicians as these infections tend to mimic HELLP (Hemolysis, Elevated Liver enzymes, and Low Platelets) syndrome and increase the chances of perinatal complications and maternal mortality. Except for tropical diseases, ever since the conception of the COVID-19 pandemic, the differentials for fever pregnancy have become extensive, and the treating clinicians need to solve the puzzle of the etiology behind these symptoms that are non-specific and might be due to both COVID-19 and tropical Infections. Prophylactic treatment for malaria is pivotal in pregnancy as immunity is decreased during pregnancy, making the patient susceptible to developing malaria-related complications. Dengue is one of the most common mosquito-borne infections found around the globe. Complications of dengue during pregnancy include pregnancy loss as well as vertical transmission of infection to the fetus. Leptospirosis, even though rare, has a wide range of complications in pregnancy ranging from fetal loss to congenital infection and oligohydramnios, thereby requiring close monitoring and prompt management during pregnancy.

We report a case series of three cases where patients presented during the period of pregnancy with fulminant hepatic failure, which turned out to be a consequence of tropical diseases. All the cases were treated successfully and discharged in stable condition.

## Introduction

Dengue is a viral disease that is mosquito-borne and has spread swiftly across the globe, especially in developing countries [1]. Dengue virus or DENV is transmitted through female mosquitoes belonging primarily to the species of *Aedes aegypti* followed by *Aedes albopictus* [2]. These mosquitoes are vectors for other diseases as well, including zika virus, yellow fever, and chikungunya. Dengue is caused by a virus of the *Flaviviridae* family and has four different serotypes that are interrelated, namely DENV-1, DENV-2, DENV-3, and DENV-4 [3]. While most aspects of dengue infection affecting health care have been discussed in detail in the literature, maternal complications as a result of dengue fever and the challenges faced during the management of dengue during pregnancy are less frequently discussed. There have been reports of dengue hemorrhagic fever and dengue shock syndrome during pregnancy, complicating the management and increasing the difficulties for clinicians [4,5]. Malaria is another tropical disease, which, according to its mortality and incidence rate, is the most serious vector-borne disease worldwide [6]. In India, more than 90% of the population resides in malaria transmission areas where the most prevalent species is *Plasmodium falciparum* followed by *Plasmodium vivax* [7]. Approximately 13 million new cases of malaria and 24,000 deaths are encountered in India annually, making malaria a concern for health care providers and policymakers [7]. India contributes 4% to the global burden of malaria and 87% to the total number of cases reported from Southeast Asia [7]. Pregnant women are specifically more susceptible to malaria infection due to the immunological changes that occur during pregnancy and predilection of *Plasmodium falciparum* to sequester in the placenta [8]. This makes pregnancy a high-risk state for malaria requiring excessive care and monitoring. Leptospirosis is a zoonosis that is caused by *Leptospira interrogans* [9]. The endemic disease occurs mainly in the tropics, with certain cases being reported from the temperate areas [9]. Presenting symptoms include fever, myalgia, rigors, nausea, vomiting, headache, dry cough, and diarrhea. Signs that can be seen in leptospirosis are hepatomegaly, splenomegaly, skin rash, aseptic meningitis, and conjunctival suffusion [10]. An atypical presentation of leptospirosis includes Weil's syndrome, which is characterized by hyperbilirubinemia, renal dysfunction, and coagulopathy.

## Case presentation

Case 1

**Figure 1:**
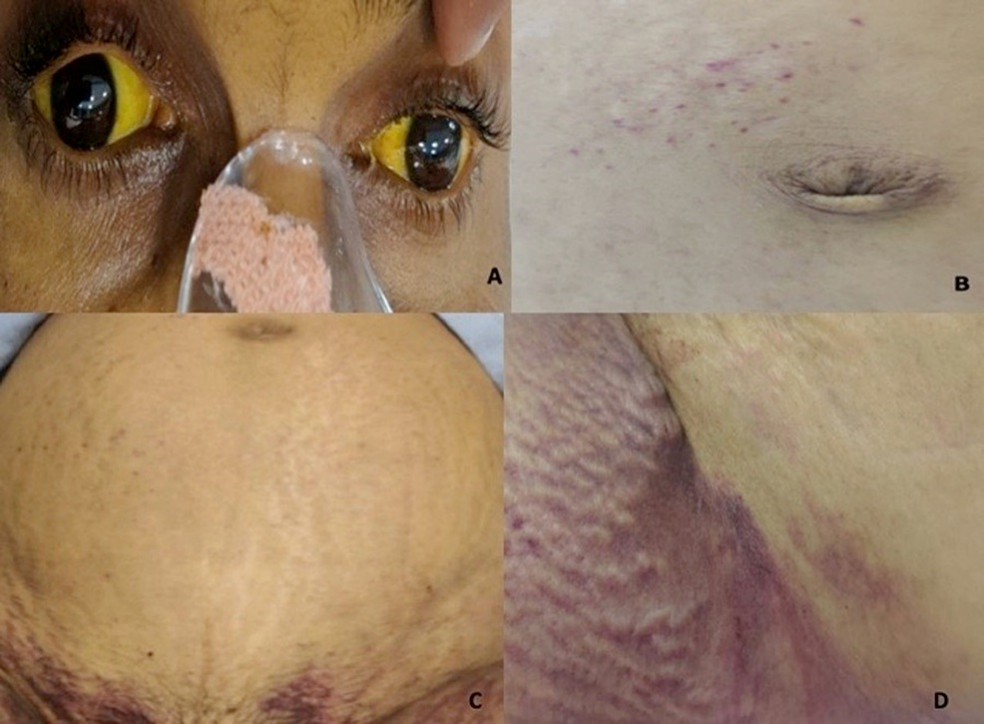
(A) Yellowish discoloration of the sclera. (B) Petechial hemorrhages over the supra-umbilical area. (C) Large petechial patch over the lower abdomen and bilateral thighs. (D) Petechial patch over the lateral aspect of the thigh.

A 33-year-old female, having three living children, presented to the emergency department on day 8 of the post-natal period with the chief complaint of fever with chills for 15 days, retro-orbital pain for 15 days, and yellowish discoloration of eyes with dark-colored urine since 10 days. There was no history of nausea, vomiting, loose stools, burning micturition, cough, or breathlessness. Obstetric history revealed three deliveries in the past, with the last delivery eight days back with a full-term male child delivered in an ambulance. She was advised admission priorly and delivered in the ambulance before reaching the peripheral healthcare center. On examination, Glasgow coma score was 4 for eyes, 5 for verbal, and 6 for motor (15/15), pulse was 92 beats per minute (regular in rhythm and volume having no special character), blood pressure was 80/50 mm of Hg in the right arm in the sitting position, and icterus was present (Figure 1A). Examination of the abdomen revealed a stretched umbilicus, yellowish discolored skin with multiple petechiae (Figure 1B); gross distention was also present with tense, shiny skin with yellowish discoloration, On palpation, the uterus could not be palpated due to gross ascites, liver and spleen could not be palpated, fluid thrill was present, and on percussion, tympanic node was heard until 5-6cm below the umbilicus and below that dull node on percussion was present, suggestive of a post-partum involuted uterus. The patient was conscious oriented with no focal neuro deficit, heart sounds were normal, and chest was bilaterally clear. On local examination, a huge area of petechiae was present on the perineum and groin (Figures 1C, 1D).

Lochia was healthy with reddish discoloration and without foul smell. Her laboratory investigations showed thrombocytopenia, raised international normalized ratio (2.11) along with increased liver enzymes, and a raised conjugated bilirubin, as shown in Table 1. The non-structural antigen for dengue fever was positive. Ultrasonography of the abdomen was performed, which showed 12.1 x 9.6 x 7.8 cm uterus suggestive of post-partum bulky changes, mild splenomegaly (12.5cm), features of acute hepatitis, and gross ascites. The patient was immediately admitted to the intensive care unit and was given intravenous fluids. In view of persistent hypotension, she was started on inotropic support. The patient was transfused a total of 10 random donor platelets and five fresh frozen plasma. The patient was managed with intravenous fluids to maintain hydration, antibiotics, injectable vitamin K, and supportive care, and she gradually improved clinically with an increase of platelet count from 17,000/dL on admission to 110,000/dL on day 6 of admission. She was finally discharged in a stable condition on day 10 of admission and is presently doing well on follow-up.

**Table 1:** Investigations of the first case RT-PCR, reverse transcription polymerase chain reaction; SARS-CoV-2, severe acute respiratory syndrome coronavirus 2

Laboratory parameter	Day 1	Day 2	Day 5	Day 7	Day 10
Hemoglobin	11.1 gm/dL	10.9 gm/dL	10.7 gm/dL	10.5 gm/dL	10.7 gm/dL
Platelet count	17,000/dL	68,000/dL	75,000/dL	110,000/dL	135,000/dL
White blood cell count	1,500/dL	23,00/dL	2,700/dL	3,200/dL	4,400/dL
Hematocrit	43	39	38	37	37
International normalized ratio	2.11	1.42	1.32	1.31	1.32
Urine routine microscopy	Positive for red blood cell				
Serum glutamic-oxaloacetic transaminase	4,600	2,100	1,670	590	110
Serum glutamic pyruvic transaminase	4,300	2,200	1,580	555	105
Total bilirubin	27.1	20.3	15.1	9.7	3.2
Unconjugated bilirubin	1.8		1.9	2.4	0.8
Conjugated bilirubin	25.3	18.4	13.2	7.3	2.4
Non-structural antigen for dengue	Positive				
RT-PCR For SARS-CoV-2	Negative				

Case 2

**Figure 2:**
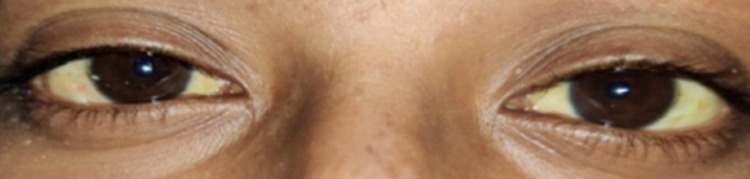
Yellowish discoloration of the sclera of case 2

A 29-year-old female primigravida with a gestational age of 39.2 weeks presented with the chief complaint of fever, which was accompanied with chills, high grade, and intermittent since 15 days, yellowish discoloration of eyes and skin since 12 days, and pain in the abdomen since 12 hours. There was no history of nausea or vomiting. On examination, the patient's pulse was 70 beats per minute (regular in rhythm and volume with no special character), blood pressure was 100/60 mm of Hg, and icterus was present, as shown in Figure 2.

On abdominal examination, the patient had yellowish discoloration of the skin over the abdomen, smiling umbilicus, striae gravidareum, and linea nigra. On palpation, the uterus was term size, and the fullness of flanks was present. Mild contractions were present. Presentation of the fetus was cephalic with regular fetal heart rate. On per vaginum examination, the cervix was favorable with dilatation of 2-3cm and effacement of 50%, station at -3, presenting part vertex with membranes forming. On cardiac auscultation, heart sounds were normal with no murmurs, and the chest was bilaterally clear on chest auscultation. The patient was conscious and oriented with no focal neurodeficit and no constructional apraxia. The patient was admitted to the intensive care unit. Laboratory investigations are given in Table 2.

**Table 2:** Laboratory investigations of case 2 RT-PCR, reverse transcription polymerase chain reaction; SARS-CoV-2, severe acute respiratory syndrome coronavirus 2

Laboratory parameter	Day 1	Day 2	Day 6
Hemoglobin	6.8 gm/dl	7.9 gm/dL (after two units of packed red cell transfusion)	9.6 gm/dL
Platelet count	1,6000/cu mm	59,000/cu mm (after five units of random donor platelet transfusion)	98,000/cu mm
White blood cell count	1,500/dL	2,300/dL	2,700/dL
Hematocrit	43	39	38
International normalized ratio	2.11	1.42	1.32
Urine routine microscopy	Negative for red blood cell		
Serum glutamic-oxaloacetic transaminase	3,500	2,320	110
Serum glutamic pyruvic transaminase	2,560	1,129	105
Total bilirubin	7.1	5.2	1.3
Unconjugated bilirubin	0.5	0.4	0.3
Conjugated bilirubin	5.6	4.8	1
Peripheral smear for malaria	Positive		
RT-PCR for SARS–CoV-2	Negative		

The patient tested positive on peripheral smear examination for *Plasmodium falciparum,* suggestive of malaria. She had raised liver enzymes and bilirubin, along with reduced hemoglobin and platelet count. She was managed with injectable artesunate, intravenous fluids, and fresh frozen plasma, and was transfused five units of platelets and two units of packed red cells. The patient had vaginal delivery on the second day of admission and delivered a female child weighing 2.23 kg. The patient improved clinically during the hospital stay and her hemoglobin and platelet count increased from 6.8 gm/dL and 16,000/cu mm, respectively, on admission to 9.6 gm/dL and 98,000/cu mm on day 6 of admission. She was discharged in stable condition on day 7 of admission and is presently doing well on follow-up.

Case 3

**Figure 3:**
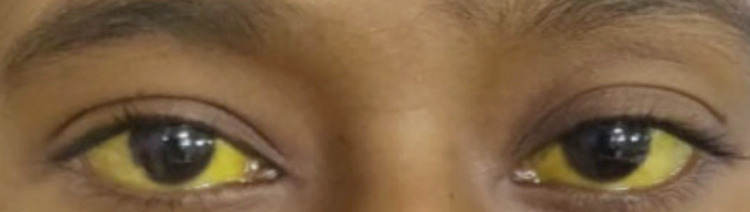
Yellowish discoloration of the sclera of case 3

A 22-year-old nulligravida with a gestational age of 38 weeks and six days presented to the emergency department with the chief complaint of fever since 10 days, yellowish discoloration of the eyes since seven days, and black-colored stools since two days. There was no history of hematemesis, nausea, or vomiting. She did not have a history of COVID-19. On examination, her pulse was 72 beats per minute (regular in rhythm), blood pressure was 90/60 mm of Hg, and icterus and pallor were present. Figure 3 shows the patient's yellowish discoloration of eyes.

On inspection of abdomen, yellowish discoloration of skin was present along with striae gravidarum and linea nigra. There were no scar marks and dilated veins. On palpation of the abdomen, the uterus was term in size with the fullness of flanks present was relaxed. Presentation of the fetus was cephalic with regular fetal heart rate. On per vaginal examination, the cervix was 2cm dilated and 20% effaced, the station was high up, and membranes were present with presenting part as the vertex. The patient was admitted to the intensive care unit. She was tested for COVID-19 through the reverse transcriptase-polymerase chain reaction method, which came out to be negative. Laboratory investigations are mentioned in Table 3.

**Table 3:** Laboratory investigations of case 3 IgM, immunoglobulin M

Laboratory parameter	Day 1	Day 2	Day 3	Day 7	Day 17
Hemoglobin	6.9 gm/dL	8.5. gm/dL	8.7 gm/dL	9.2 gm/dL	9.7 gm/dL
Platelet count	15,000/dL	40,000/dL	56,000/dL	99,000/dL	117,000/dL
White blood cell count	16,500/dL	17,600/dL	15,300/dL	9,800/dL	7,600/dL
International normalized ratio	2.5	1.98	1.5	1.43	1.33
Urine routine microscopy	Positive for red blood cell				
Serum glutamic-oxaloacetic transaminase	2,700	2,200	1,320	320	76
Serum glutamic pyruvic transaminase	2,400	2,150	1,280	355	68
Total bilirubin	21.3	20.3	17.1	13.7	2.5
Unconjugated bilirubin	1.8	2.4	0.9	0.9	0.3
Conjugated bilirubin	19.5	17.9	16.2	12.6	2.2
IgM for leptospirosis	Positive				

She was tested for IgM for leptospirosis in view of fever, which was positive. The patient had thrombocytopenia along with anemia in view of which she was transfused one unit of packed red cells and five units of random donor platelets. The patient was managed with injectable doxycycline, fresh frozen plasma, injectable vitamin K, and intravenous fluids. During the course of the hospital stay, the patient went into labor and delivered a male child weighing 1.9 kg on day 3 of admission through vaginal delivery who was shifted to neonatal intensive care unit and was started on injectable gentamycin and ampicillin. Breastfeeding was deferred as the patient was on doxycycline. During the course of the hospital stay, both the mother and child improved clinically, and the mother’s platelet count increased with a decrease in liver enzymes. The patient and her child were discharged after 17 days of admission in stable condition and are presently doing well on follow-up.

## Discussion

Tropical infections during the perinatal period are of substantial importance primarily in developing nations like India. Detecting these tropical infections early in the course along with prompt management measures may help in preventing maternal mortality. The first case reported here was of dengue-induced fulminant hepatitis in a pregnant female occurring in the third trimester. It is postulated that pregnancy predisposes the patient to develop dengue hemorrhagic fever, and due to the physiological changes of pregnancy leading to hemoconcentration, diagnosis of dengue becomes difficult for the clinicians during pregnancy [11]. A meta-analysis that studied the effect of infections on pregnancy showed that viral infections and parasitic infections increase the risk of pre-eclampsia in pregnancy by causing endothelial dysfunction [12]. In the case of dengue in pregnancy reported in this case series, there was a development of fulminant hepatic failure. The possible mechanism for the insult to the liver in the case of dengue fever is due to the direct effect of the virus or due to the immune response exerted by the host on liver cells [13]. Circulatory compromise, hypoxia as a result of shock, and metabolic acidosis are other postulated mechanisms of dengue-induced hepatic failure [13]. Treatment of dengue fever in our case was carried out in the form of intravenous fluid resuscitation along with platelet transfusion. An important aspect of this case of dengue in pregnancy was the late presentation to the health care facility and an ambulance delivery. Being in the rural central India, such a case warns against the complications that might arise due to lack of knowledge and awareness among the pregnant females regarding the requirement of proper timely health care check-ups and reporting to a health care center even for benign-looking symptoms such as fever, which was ignored by the patient. The health policy makers need to target such aspect of the society who are unwarned and unware of the possible complications of pregnancy and the effect of endemic tropical diseases on pregnancy.

The second case reported was of malaria in pregnancy leading to fulminant hepatic failure. Malaria in pregnancy, which has been reported in one of our cases, is a serious issue for the clinicians of India. Malaria infection during pregnancy is associated with maternal anemia as well as low birth weight and premature delivery [14]. However, in certain cases, such as the one reported in this case series, malaria can lead to life-threatening complications such as fulminant hepatitis. Patients with malaria developing fulminant hepatitis have a mortality rate of 40% [15]. The postulated mechanism to suggest the cause of liver injury is that there is ischemia that occurs due to an alteration of blood flow through the liver [15]. This alteration is due to adherence of infected red blood cells to the endothelial cells, which results in blocking of the sinusoids. Early diagnosis, such as in the reported case, and treatment of malaria aid in rapid reversal of liver dysfunction. *Plasmodium falciparum* is known to sequester in the maternal blood spaces present in the placenta [16]. By doing so, it avoids clearance through the spleen and other immune cells. *Plasmodium falciparum* expresses VAR2CSA on the red blood cells, which is a protein that sticks to the chondroitin sulphate, which is a receptor present in the placenta [17]. A recent trial has shown that vaccines against VAR2CSA known by the name of PRIMVAC have the potential to prevent placental malaria [18]. This vaccination can be a game-changer for countries combatting malaria during pregnancy. Various trials have formulated artemisinin-based combination treatments (ACTs) as the first-line drugs against malaria in the second and third trimesters of pregnancy, which showed promising results in the reported case too [19].

The third case reported was of Weil’s syndrome in pregnancy. Weil’s syndrome associated with leptospirosis is characterized by jaundice, renal failure, and coagulopathy [20]. The pathophysiology behind Weil's syndrome is leptospirosis leading to damage to the small blood vessels resulting in vasculitis and massive migration of intravascular fluid to the interstitial compartment [20]. Both of these mechanisms along with the direct cytotoxic effects of *Leptospira* lead to renal and hepatic injury. Leptospirosis in pregnancy is rather uncommon, with only a few cases being reported [21]. Unusual presentation of leptospirosis with features mimicking pregnancy-related liver disease such as the case described above has been reported before [21]. Leptospirosis can lead to adverse fetal and maternal outcomes such as fetal demise, congenital infection, and oligohydramnios. Carles et al. had reported a case series where the risk of abortion in leptospirosis was more than 50% [22]. Our patient was managed successfully with penicillin G, platelet transfusion, and other supportive measures. Coagulopathy induced due to liver failure in all the reported cases was treated successfully by injectable vitamin K and fresh frozen plasma transfusion. This shows that prompt diagnosis and resuscitation of fulminant hepatic failure even in the perinatal period may help prevent maternal and neonatal mortality. All three reported cases recovered completely with no fetal complications and are presently doing well on follow-up.

An important aspect of our case series is the differential of HELLP (Hemolysis, Elevated Liver enzymes, and Low Platelets) syndrome because of overlapping features of HELLP and fulminant hepatitis due to tropical diseases in pregnancy. While there was a history of fever in all three cases with evidence of seropositivity for tropical infections along with an increase in conjugated bilirubin independently, it makes the diagnosis of HELLP syndrome less likely. In the above scenario, it is plausible to conclude that tropical infections were the etiology behind the fulminant hepatic failure in pregnancy. Diagnosing and managing such tropical infections in pregnancy during the COVID-19 era is another challenge that was faced during the management of our cases. COVID-19 has also been reported in association with HELLP syndrome with similar presenting features such as our cases [23,24]. COVID-19 and dengue co-infection have also been reported before leading to adverse outcomes due to antibody-dependent enhancement, which also led to hepatic failure [25]. However, an RT-PCR was conducted for all three cases, which were negative, thus ruling out SARS-CoV-2 as the probable cause of fever and hepatic failure.

## Conclusions

Hence, we conclude that tropical diseases such as malaria and dengue may complicate the course of pregnancy through complications such as fulminant hepatic failure, which, if treated in time, can lead to favorable outcomes. Therefore, tropical diseases need to be considered as an important differential by the clinicians for any pregnant female presenting with hepatic failure in order to ensure an early diagnosis and prompt management to prevent maternal mortality. The outcome of such demanding infections requires a multidisciplinary approach with proper coordination among obstetricians, physicians, and pathologists, along with appropriate intensive care support.
